# Functional Integrity of the Inferior Vestibular Nerve and Posterior Canal BPPV

**DOI:** 10.3389/fneur.2020.00894

**Published:** 2020-08-26

**Authors:** Avi Shupak, Rohi Falah, Margalith Kaminer

**Affiliations:** ^1^Unit of Otoneurology, Lin Medical Center, Haifa, Israel; ^2^Rappaport Faculty of Medicine, Technion Israel Institute of Technology, Haifa, Israel; ^3^Faculty of Social Welfare and Health Sciences, University of Haifa, Haifa, Israel; ^4^Department of Otolaryngology Head and Neck Surgery, Galilee Medical Center, Nahariya, Israel

**Keywords:** cervical evoked myogenic potentials, vestibular nerve, vertigo, benign paroxysmal positional, saccule and utricle, semicircular canals, surveys and questionnaires, caloric tests

## Abstract

The functional integrity of the inferior vestibular nerve (IVN) may be evaluated by the cervical vestibular evoked myogenic potential (cVEMP) response, which requires signal transmission via the nerve. As functional integrity of the IVN innervating the posterior semicircular canal is required to produce the typical positioning vertigo and nystagmus characterizing posterior canal benign paroxysmal positional vertigo (PCBPPV), we hypothesized that normal cVEMPs would be found in most PCBPPV patients. Twenty-four PCBPPV patients participated in a prospective cohort study. All were treated by canal repositioning maneuver and had air-conduction cVEMP and videonystagmography (VNG). Follow-up evaluations including history and otoneurological bedside examination were carried out 1, 3, 6, and 12 months after the initial treatment. At the last follow-up, the patients filled the Dizziness Handicap Inventory (DHI) questionnaire. Normal cVEMPs were recorded in 19 (79%) and were absent in 5 (21%) of the subjects. The average DHI in the patients with normal cVEMP was 16.42 ± 17.99 vs. 0.4 ± 0.89 among those with pathological cVEMP (*p* < 0.04, Mann–Whitney test). Thirteen (54%) patients experienced recurrent PCBPPV (rPCBPPV). The average DHI score was significantly higher among patients having recurrence (22.15 ± 18.61) when compared to those with complete cure (2.36 ± 5.98; *p* < 0.003, Mann–Whitney test). Ten (77%) of the subjects with rPCBPPV had normal and 3 (23%) had pathological cVEMP as compared to 9 (82%) and 2 (18%) subjects in the non-recurrent (nrPCBPPV) group (Fisher's exact test—not significant). cVEMP p13 and n23 wave latencies and amplitudes, inter-aural differences in p13-n23 peak-to-peak amplitudes, and response thresholds did not differ between the groups. No differences were found between the rPCBBPV and nrPCBBPV groups in VNG caloric lateralization and directional preponderance values. We have found that in most cases, PCBPPV symptoms and signs are associated with normal cVEMP response supporting the role of IVN functional integrity. The absent cVEMPs in the minority of patients, although having similar clinical presentation, raise the possibility that the ipsilateral saccule is affected by the same pathology causing degeneration of the utricle macula. Alternatively, lacking inhibitory stimuli from the involved ipsilateral utricle or partial degeneration of the IVN and ganglion could explain the diminished cVEMP response.

**Clinical Trial Registration**: The study was registered in ClinicalTrials.gov Internet site (study ID—NCT01004913; https://clinicaltrials.gov/ct2/show/NCT01004913?cond=BPPV&cntry=IL&draw=2&rank=3).

## Introduction

Benign paroxysmal positional vertigo (BPPV) is the most common peripheral cause of vertigo. Lifetime prevalence is estimated to be 2.4% (1), and 20–25% of patients referred to dizziness/vertigo centers are diagnosed as suffering from BPPV (2, 3).

Current understanding of posterior semicircular canal benign paroxysmal positional vertigo (PCBPPV) pathogenesis involves the dislodgement of otoconial debris detached from the utricle into the posterior semicircular canal (PSCC). The effect of the gravitational forces on these debris leads to deflection of the canal cupula, resulting in vestibular afferent firing transmitted via the inferior vestibular nerve to the vestibular nuclei (4). The dependence of PCBPPV symptoms and signs on the integrity of PSCC innervation is demonstrated by its complete resolution following singular neurectomy in reluctant cases (5).

The cervical vestibular evoked myogenic potentials (cVEMPs) are short-latency electromyographic responses that can be recorded from the ipsilateral sternocleidomastoid muscle (SCM) during its contraction phase in response to air and bone-conducted acoustic stimuli, skull tapping, and galvanic stimulation (6). The cVEMP pathway is believed to originate in the saccular macula and continues through the ipsilateral inferior vestibular nerve and ganglion, vestibular nucleus, ipsilateral vestibulospinal tracts, spinal motor nucleus, and the sternocleidomastoid muscle. This sacculo-collic reflex is characterized by biphasic waves with initial positivity (p13) followed by a negative wave (n23) (6, 7). As the cVEMP response of the sacculo-collic reflex depends on the spreading of neural signals via the inferior vestibular nerve, it has been suggested that cVEMPs would be preserved in patients having the clinical presentation of PCBPPV (8).

The aim of the study was to examine cVEMP response in patients suffering from PCBPPV. Our hypothesis was that cVEMP would be recorded in most patients suffering from PCBPPV.

## Patients and Methods

### Sample and Design

Twenty-four consecutive patients suffering from PCBPPV (10 males, 14 females) aged 32–60 years (mean 51.8 ± 7.36 years; median 54.5 years) referred to a tertiary otoneurology unit were recruited to a prospective cohort study. PCBPPV was diagnosed by a Dix-Hallpike maneuver demonstrating crescendo–decrescendo geotropic rotatory nystagmus with an upbeating vertical component, which changed its direction when the patient resumed sitting position. The upper age limit of 60 years was elected in order to avoid potential bias due to the known deterioration in cVEMP response in older individuals (9). After signing an informed consent, the patients had baseline evaluation that included detailed history with emphasis on previous or existing ear disease, complete otoneurological bedside examination including microscopic otoscopy, eye-movement examination with and without Frenzel glasses, post-head shaking test, head impulse test, supine roll test, Dix-Hallpike maneuver, enhanced Romberg test, tandem walking test, and Fukuda stepping test.

Following the diagnosis of PCBPPV, treatment was completed by Epley's canalith repositioning procedure (CRP) (10). After an interval of 30 min, a second Dix-Hallpike maneuver was carried out and Epley's CRP was repeated as required. All patients in our cohort had negative findings on Dix-Hallpike maneuver following a maximum of two Epley's CRPs.

All participants had the following laboratory evaluation the days following successful CRP: pure tone, speech and impedance audiometry; videonystagmography (VNG) including tests for oculomotor system integrity (saccadic, gaze, optokinetic, and pursuit systems), tests for spontaneous, positional, and positioning nystagmus (Dix-Hallpike maneuver), and the alternate binaural bithermal caloric test (11); and cVEMPs testing including p13-n23 wave recordings and response threshold.

The study participants met the following inclusion criteria: (1) age 18–60 years; (2) negative history for concurrent or previous otological disease beside positional vertigo; (3) Dix-Hallpike maneuver positive for the presence of unilateral PCBPPV; (4) normal air-conduction pure tone, speech, and impedance audiometry; and (5) normal VNG test battery findings or compatible with peripheral vestibulopathy alone.

Follow-up evaluations including history and otoneurological bedside examination were conducted 1, 3, 6, and 12 months after the initial treatment. On the 12-months follow-up appointment, the patients filled the Dizziness Handicap Inventory (DHI) questionnaire (12).

The study protocol and procedures were approved by the committee for human experiments, Meir Medical Center, Kfar Saba, Israel, and were registered in ClinicalTrials.gov Internet site (study ID—NCT01004913; https://clinicaltrials.gov/ct2/show/NCT01004913?cond=BPPV&cntry=IL&draw=2&rank=3). All subjects gave written informed consent in accordance with the Declaration of Helsinki.

### Cervical Vestibular Evoked Myogenic Potentials (cVEMP)

cVEMPs were performed bilaterally using the Navigator Pro System (Bio-Logic Systems Corp., Mundelein, IL, USA). Muscle activity was recorded in the supine position with the subject lying using Ag/AgCl electrodes. The active electrode was attached over the main bulk of the SCM muscle, approximately half the distance between the mastoid tip and the sternal notch. A reference electrode was placed over the upper sternum and the ground electrode on the forehead. Tone-burst air stimuli were presented to the ears through insert earphones at 4.3 Hz with a central frequency of 500 Hz. To achieve enough contraction of the SCM, subjects were instructed to lift their heads. Electromyographic activity was recorded simultaneously from both sides to minimize possible effects due to asymmetric muscle tone. The time window for recording was 53.3 ms; the electromyographic potential was amplified ×1,000 and filtered to the 10–1,500-Hz frequency range. Each cVEMP response was the average of the responses to 200 consequent stimuli. The eligibility criterion was correlation above 0.75 for two successive responses and p13-n23 peak-to-peak amplitude at least twice the size of the pre-stimulation baseline recording (13). Initial stimuli were provided at 90 dBHL decreasing in 5 dBHL steps. The cVEMP threshold was determined at the lowest stimulus level, still producing a response. Whenever a response could not be elicited at 90 dBHL, stimulus increase up to a maximal level of 97 dBHL was allowed. When a response could not be obtained at that level, the cVEMP was defined absent.

The following cVEMP parameters were measured: p13 and n23 wave latencies and amplitudes; p13-n23 peak-to-peak amplitude; inter-aural amplitude difference (IAD) defined as the ratio between the right and left peak-to-peak amplitude difference and the sum of both sides' peak-to-peak amplitude; and response threshold.

### Statistical Analysis

cVEMP was defined as abnormal for IAD >35% or absent response (14). Caloric test results showing unilateral weakness >25% or directional preponderance >30% were considered pathological (15).

The proportions of abnormal cVEMP and caloric test results were compared between the patients who suffered PCBPPV recurrences during the 12-months follow-up period (rPCBPPV) and those having complete resolution (nrPCBBPV) employing Fisher's exact test.

cVEMP wave latencies, peak-to-peak amplitudes, IAD, and thresholds were compared between the rPCBBPV and nrPCBPPV groups by the Student unpaired two-tailed test or the non-parametric Mann–Whitney test according to the Shapiro–Wilk normality test results.

DHI questionnaire results were compared using the Mann–Whitney test.

*P*-values <0.05 were considered statistically significant. Statistical analysis was performed using the GraphPad InStat version 3.06 software (San Diego, CA, USA).

## Results

Normal cVEMPs were recorded in 19 (79%) and were absent in 5 (21%) of the subjects. In all absent cVEMP cases, the missing response was ipsilateral to the PCBBPV side. None of the bilaterally elicited cVEMPs met the criteria of IAD >35%.

**Figure 1:**
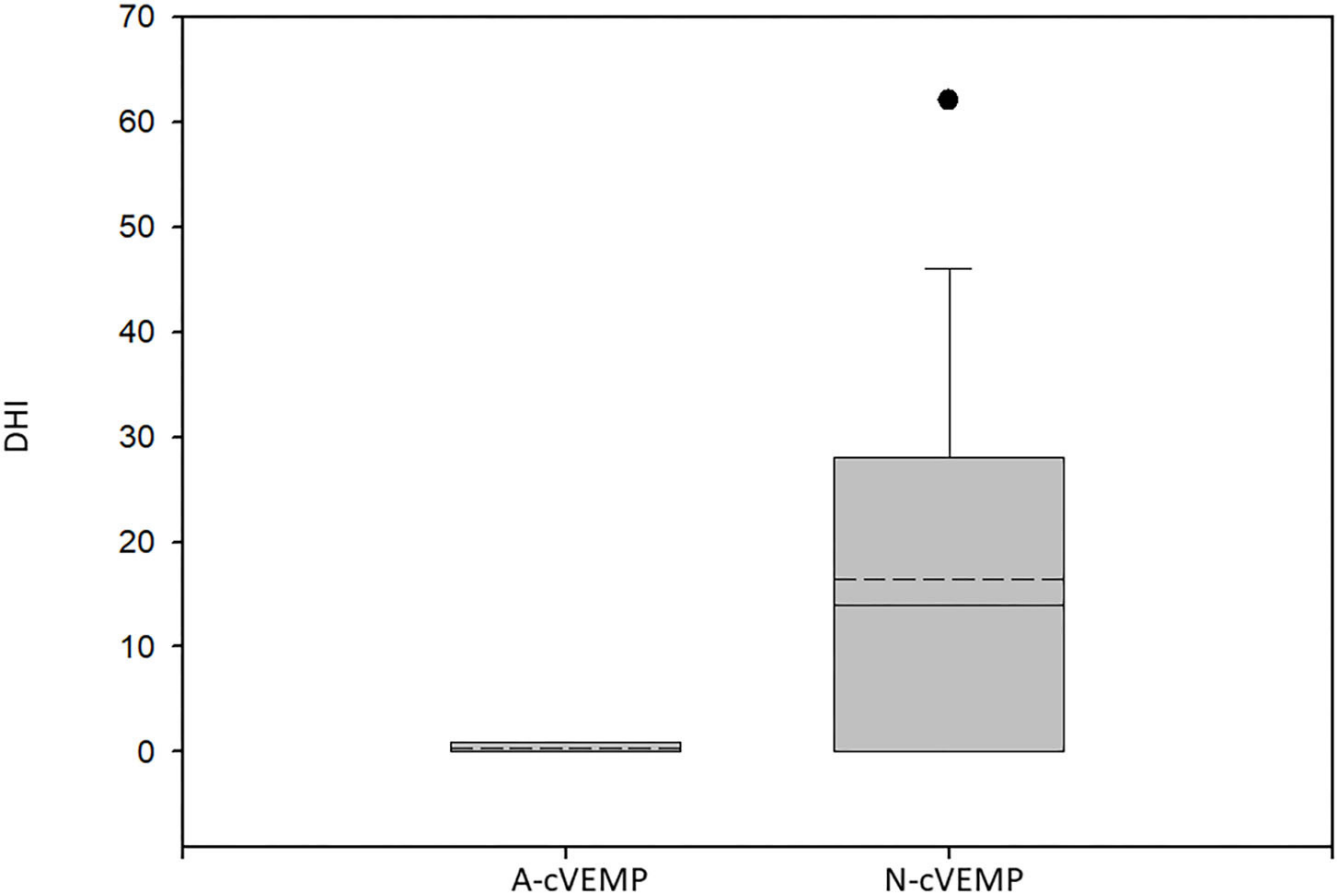
Box plot of the Dizziness Handicap Inventory scores of patients with absent cVEMPs and those with normal responses. A-cVEMP, absent cVEMPs; N-cVEMP, normal cVEMPs; DHI, Dizziness Handicap Inventory score. The boundary of the box closest to zero indicates the 25th percentile, the solid line within the box marks the median, the dashed line marks the mean, and the boundary of the box farthest from zero indicates the 75th percentile. Whiskers above and below the box indicate the 90th and 10th percentiles. Circles above and below the 90th and 10th percentiles mark outlying data points. Significantly lower scores were found for the patients with missing cVEMPs (*p* < 0.04, Mann–Whitney test).

The mean DHI score at 12 months from diagnosis in patients with normal cVEMP was 16.42 ± 17.99 vs. 0.4 ± 0.89 among those with absent cVEMP (*p* < 0.04, Mann–Whitney test) (Figure 1).

VNG was performed in 19 (79%) of the patients, and pathological caloric test results were found in 6 (32%). Five had significant caloric lateralization (>25%) and 1 increased directional preponderance (>30%). Canal paresis was ipsilateral to the PCBPPV side in all cases while the directional preponderance of the caloric nystagmus slow phase velocity was to the contralateral side.

During the 1-year follow-up 13 (54%) patients experienced rPCBPPV.

Ten (77%) of the subjects with rPCBPPV had normal and 3 (23%) pathological cVEMPs as compared to 9 (82%) and 2 (18%) subjects in the nrPCBPPV group. The proportions of absent cVEMPs did not differ between the groups (Fisher's exact test).

The variance in p13 and n23 wave latencies, p13-n23 peak-to-peak amplitudes, IAD percentage, and cVEMP thresholds could not predict PCBPPV recurrences (Table 1).

**Table 1:** cVEMP wave latencies and amplitudes, p13-n23 peak-to-peak amplitudes, response thresholds, and inter-aural amplitude differences (IAD) (mean ± standard deviation) compared between the patients with recurrent posterior canal BPPV (rPCBPPV) and patients with no recurrences (nrPCBPPV).

	**rPCBBPV**	**nrPCBPPV**	**Statistical significance**
**RIGHT EAR**			
p13 latency (ms)	15.01 + 1.72	14.76 + 1.01	NS (unpaired *t*-test)
n23 latency (ms)	23.76 + 2.37	23.03 + 1.76	NS (unpaired *t*-test)
p13-n23 peak-to-peak amplitude (μV)	94.68 + 36.45	84.82 + 54.75	NS (unpaired *t*-test)
Threshold (dBHL)	90.83 + 5.15	88.75 + 5.17	NS (Mann–Whitney)
**LEFT EAR**			
p13 latency (ms)	15.19 + 3.17	15.04 + 1.24	NS (Mann–Whitney)
n23 latency (ms)	23.35 + 2.03	23.09 + 1.28	NS (unpaired *t*-test)
p13-n23 Peak-to-peak amplitude (μV)	89.49 + 35.07	74.46 + 33.96	NS (unpaired *t*-test)
Threshold (dBHL)	90.45 + 5.22	89.37 + 4.95	NS (Mann–Whitney)
IAD (%)	11.74 + 11.2	13.58 + 5.5	NS (unpaired *t*-test)

VNG was conducted in 8 of the patients with rPCBPPV. Pathological caloric test was found in 3 (38%) of them as compared to 3 of 11 patients (27%) of the nrPCBBPV group (Fisher's exact test—not significant). No significant differences were found between the rPCBBPV and nrPCBBPV groups in VNG caloric lateralization and directional preponderance values (Table 2).

**Table 2:** Videonystagmography caloric tests results compared between the patients with recurrent posterior canal BPPV (rPCBPPV) and patients with no recurrences (nrPCBPPV).

	**rPCBBPV**	**nrPCBPPV**	**Statistical significance**
Lateralization (%)	4.25 + 35.1	13.7 + 25.27	NS (Mann–Whitney)
Directional preponderance (%)	11 + 17.47	2 + 17.52	NS (unpaired *t*-test)

The average DHI in patients with pathological caloric tests was 19.33 ± 17.46 vs. 11 ± 17.16 among the patients that had normal results. This difference did not reach statistical significance (Mann–Whitney test).

**Figure 2:**
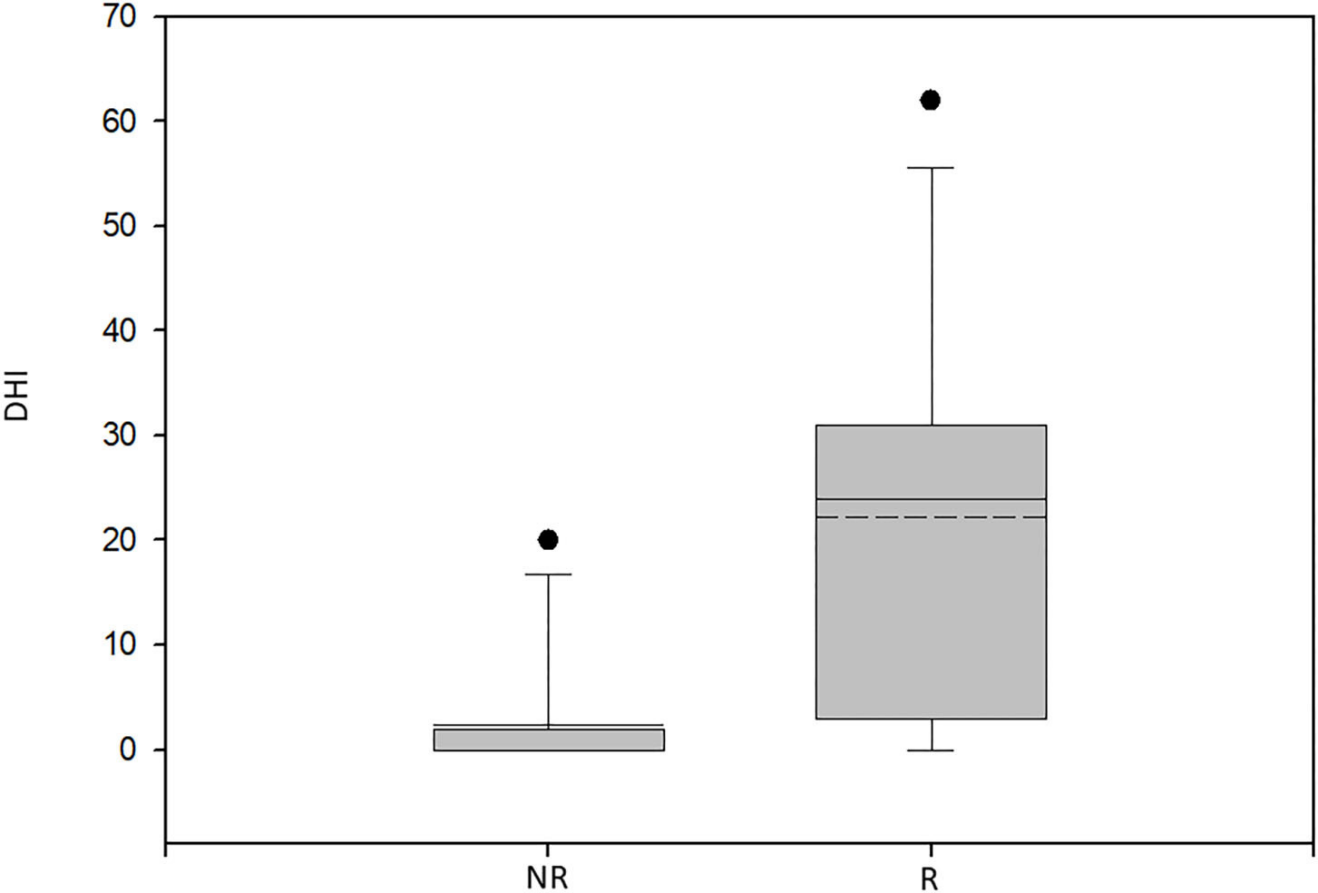
Box plot of the Dizziness Handicap Inventory scores for the patients with recurrent BPPV and non-recurrent BPPV. NR, non-recurrent group; R, recurrent group; DHI, Dizziness Handicap Inventory score. Significantly lower scores were found for the non-recurrent group (*p* < 0.003, Mann–Whitney test).

The average DHI score 12 months post-presentation was significantly higher among patients having recurrences (22.15 ± 18.61) when compared to those with complete resolution (2.36 ± 5.98; *p* < 0.003, Mann–Whitney test) (Figure 2).

## Discussion

Although cVEMPs could be recorded in most PCBPPV patients (79%), the elicited response was missing in 5 (21%) despite the presence of characteristic clinical presentation. Functioning neural pathways transmitting the provoked signal from the PSCC ampullary crest via the inferior vestibular nerve to the medial vestibular nucleus is required for the full clinical presentation of PCBPPV to evolve. The failed cVEMP response might be explained by involvement of organs that contribute to the sacculo-collic reflex arch but with no effect on the PSCC-inferior vestibular nerve pathway. Similarly, PSCC dysfunctions have been registered with rotation test and video-head impulse testing in patients developing PCBPPV despite reduced vestibulo-ocular reflex gain for the mild and high-frequency domains, likely due to a transient canal disorder (16–18).

One possible explanation is saccular dysfunction. The utricle and saccule maculae have similar anatomic characteristics and may be affected by the same pathological process. As functional saccule is required for the cVEMP response, degeneration of this end organ or its innervation would result in pathological cVEMP. Support for this reasoning is provided by the histopathological observation of ganglion cell loss in the saccular nerves of temporal bones from BPPV patients (19).

Another possibility is partial derangement of the inferior vestibular nerve still transmitting the canal afferent signals initiating the eye-movement and vertigo symptoms although hampering cVEMP response. Previous BPPV-related anatomical studies have reported 30–50% loss of inferior vestibular nerve neurons and degenerative changes in the inferior vestibular ganglion (19, 20). In this context, it is of interest that the mean DHI score of the patients with no cVEMP response was significantly lower than that of those with normal cVEMPs. The reduction in PCBPPV symptoms, and accompanied emotional and physical impact, which are evaluated by the DHI questionnaire, might be explained by a decrease in the transmission of the offending signals secondary to the anatomical changes described.

A limitation of the study involves the conduction of cVEMP by air conduction alone. Although the inclusion criteria precluded conductive hearing loss, it is argued that cVEMPs can be elicited by bone stimulation when air-conduction response fails albeit normal air-conduction audiometry.

Further limitation is the relatively small size of our cohort requiring a larger-scale study supporting our results.

Whereas cVEMPs test type-I vestibular hair cells located at the peri-striolar region of the saccule, subjective visual vertical (SVV) represents a test assessing regular afferents coming from more peripheral saccular regions. Testing SVV might have disclosed functional peripheral saccular regions in the face of missing cVEMP response (21).

Animal studies showed that both saccule and utricle have inhibitory projections to the ipsilateral SCM whereas the utricle has an additional excitatory projection to the contralateral SCM (22). It was estimated that the air-conducted cVEMP response is composed of 74 and 26% saccular and utricular components, respectively (23). Degeneration of the utricle macula, superior vestibular nerve, and ganglion were repeatedly described in PCBPPV (19, 20, 24, 25). Thus, reduced contribution of involved ipsilateral utricle to the cVEMP response might explain its observed absence among some PCBPPV patients.

Although the aim of our study was the examination of cVEMP in PCBPPV patients, ocular VEMP responses (oVEMPs) could have contributed to the delineation and extent of utricular involvement in our patients (26–28).

It has also been suggested that the otolithic organs exert inhibitory signals on the PSCC excitatory activity converging in the medial vestibular nucleus (19, 29). Thus, otolith dysfunction as reflected by pathological cVEMP might even contribute to the clinical presentation of PCBPPV (30–32).

While a recent study did not find differences in any of the cVEMP parameters between PCBPPV patients and matching healthy controls (27), most previous publications have reported rates of abnormal cVEMPS within the range of 23.5–39% (4, 30, 33–40). The higher occurrence of pathological cVEMP previously found might stem from the different criteria employed. While in ours and other studies (26) the normalized criterion of increased IAD and missing cVEMP responses were the parameters taken into consideration, others used in addition the less conservative criteria of prolonged wave latencies and decreased amplitudes (30, 33–40). Also, two of the studies (33, 39) included lateral and anterior canal variants of BPPV while the reported cVEMP results did not distinguish between the groups. As p13-n23 wave latencies and amplitudes carry high intersubject and intrasubject variability (30, 41), we preferred to use the normalized parameter of IAD and qualitative approach defining cVEMP response as either present or absent.

In contradiction to ours and others' results demonstrating pathological cVEMP findings among PCBPPV patients, two previous studies with a limited number of patients found normal p13-n23 potentials in all their subjects. Murofushi et al. (8) reviewed cVEMP findings in 47 vestibular neuritis patients, 10 of which developed PCBPPV. While cVEMP response was missing in 16 (34%) of the patients implying inferior vestibular nerve involvement, it was present in all their 10 patients suffering from PCBPPV. Heide et al. (42) described three additional patients with normal cVEMPs.

Accumulating data suggest that utricular dysfunction as evaluated by oVEMPs is the main counterpart of PCBBPV while cVEMP response is more often preserved (27, 28). This supports the current understanding of PCBBPV pathogenesis involving dislodgement of otoconial debris detached from the utricle into the underlying PSCC.

The study patients were followed up for 12 months, which is the time frame in which most BPPV recurrences are anticipated (43). The rate of rPCBPPV in our cohort was 54%, higher than the 0–18% recurrence rates previously reported for the 1-year follow-up (44, 45). Although our cohort included patients suffering from isolated BPPV with no concomitant or previously diagnosed inner ear disease (primary BPPV), the presence of subclinical vestibulopathy is still a possibility. This might be reflected by the pathological caloric test results in 32% of the patients having VNG, indicating ipsilateral horizontal semicircular canal dysfunction, and absent cVEMPs in 21% implying underlying saccular or sacculo-collic pathway dysfunction. As otological comorbidities carry a higher risk for the development and recurrence of BPPV (46, 47), subclinical vestibulopathy might explain the high recurrence rate among our patients. The DHI scores of the rPCPPPV group at the end of the 12-months follow-up were significantly higher in accordance with continuous suffering due to the continuous positional vertigo.

Previous studies reported that abnormal or absent cVEMPs among PCBPPV patients were related to higher incidence of recurrence as well as to increased resistance to treatment and larger number of canalith-repositioning maneuvers required toward remission (30, 31, 39). We and others (26, 37) could not support this last notion, as the rate of absent cVEMP, wave latencies, p13-n23 amplitudes, IAD values, and response thresholds were similar in the rPCBPPV and nrPCBPPV groups and all patients in our cohort recovered following 1–2 CRPs.

## Data Availability Statement

The raw data supporting the conclusions of this article will be made available by the authors, without undue reservation.

## Ethics Statement

The studies involving human participants were reviewed and approved by Committee for human experiments, Meir Medical Center, Kfar Saba, Israel. The patients/participants provided their written informed consent to participate in this study.

## Author Contributions

AS conceived and designed the study, analyzed and interpreted the results, and wrote the manuscript. RF collected the data, organized the database, and revised the manuscript. MK performed the tests and revised the manuscript. All authors approved the final version of the text.

## Conflict of Interest

The authors declare that the research was conducted in the absence of any commercial or financial relationships that could be construed as a potential conflict of interest.
